# Characterisation of Development and Electrophysiological Mechanisms Underlying Rhythmicity of the Avian Lymph Heart

**DOI:** 10.1371/journal.pone.0166428

**Published:** 2016-12-08

**Authors:** Sajjida Jaffer, Petr Valasek, Graham Luke, Munirah Batarfi, Benjamin Jason Whalley, Ketan Patel

**Affiliations:** 1 School of Biological Sciences, University of Reading, Whiteknights, Reading, United Kingdom; 2 School of Chemistry, Food and Nutritional Sciences and Pharmacy, University of Reading, Whiteknights, Reading, United Kingdom; USF Health Morsani College of Medicine, UNITED STATES

## Abstract

Despite significant advances in tissue engineering such as the use of scaffolds, bioreactors and pluripotent stem cells, effective cardiac tissue engineering for therapeutic purposes has remained a largely intractable challenge. For this area to capitalise on such advances, a novel approach may be to unravel the physiological mechanisms underlying the development of tissues that exhibit rhythmic contraction yet do not originate from the cardiac lineage. Considerable attention has been focused on the physiology of the avian lymph heart, a discrete organ with skeletal muscle origins yet which displays pacemaker properties normally only found in the heart. A functional lymph heart is essential for avian survival and growth *in ovo*. The histological nature of the lymph heart is similar to skeletal muscle although molecular and bioelectrical characterisation during development to assess mechanisms that contribute towards lymph heart contractile rhythmicity have not been undertaken. A better understanding of these processes may provide exploitable insights for therapeutic rhythmically contractile tissue engineering approaches in this area of significant unmet clinical need. Here, using molecular and electrophysiological approaches, we describe the molecular development of the lymph heart to understand how this skeletal muscle becomes fully functional during discrete *in ovo* stages of development. Our results show that the lymph heart does not follow the normal transitional programme of myogenesis as documented in most skeletal muscle, but instead develops through a concurrent programme of precursor expansion, commitment to myogenesis and functional differentiation which offers a mechanistic explanation for its rapid development. Extracellular electrophysiological field potential recordings revealed that the peak-to-peak amplitude of electrically evoked local field potentials elicited from isolated lymph heart were significantly reduced by treatment with carbachol; an effect that could be fully reversed by atropine. Moreover, nifedipine and cyclopiazonic acid both significantly reduced peak-to-peak local field potential amplitude. Optical recordings of lymph heart showed that the organ’s rhythmicity can be blocked by the HCN channel blocker, ZD7288; an effect also associated with a significant reduction in peak-to-peak local field potential amplitude. Additionally, we also show that isoforms of HCN channels are expressed in avian lymph heart. These results demonstrate that cholinergic signalling and L-type Ca^2+^ channels are important in excitation and contraction coupling, while HCN channels contribute to maintenance of lymph heart rhythmicity.

## Introduction

The lymph heart is a small oval, hollow organ, the contractions of which assist the flow of lymph. In fish, reptiles, amphibians and birds, the lymph heart actively pumps lymph throughout the lymphatic system and into the veins [1]. In many birds, lymph hearts are functional only during embryonic life and, after hatching, degenerate since their function is superseded by skeletal muscle contractions and respiration-induced pressure fluctuations [2]. In contrast, in amphibians, several lymph hearts are present that remain functional into adult life [1].

As such, study of the molecular and functional features underlying the unique behaviours of the avian lymph heart’s development, rhythmicity and termination could offer useful and exploitable insights into therapeutic tissue engineering. In this regard, cardiac tissue engineering remains a significant challenge with heart disease accounting for ~40% of all deaths [3] and heart transplant, with its accompanying challenges, representing the only treatment approach for end stage heart disease patients [4]. While experimental advances, such as the identification of potential progenitor cell populations in cardiac tissue and the development of biocompatible scaffolds and bioreactors that could support organ growth [5–7] have been made in recent years, a detailed understanding of the unique molecular mechanisms at play during development that give rise to tissues with functionally rhythmic properties remain relatively poorly understood [8].

Avian lymph heart comprises three layers: an inner endothelial cell layer lining the organ’s cavity, an outer layer of fibrous connective tissue that facilitates attachment of the lymph heart to the vertebral column and a middle layer of muscle fibres which are highly branched and of smaller diameter reminiscent of cardiac muscle [9–11] Despite the cardiac similarity of myotubes, lymph hearts only express skeletal muscle markers [10] and not determinants of cardiac tissue, such as *GATA-4*, *-5* and *-6*, and cardiac *troponin* [11]. Nevertheless, the organ exhibits spontaneous, rhythmic contractions [10–13]. Thus, the lymph heart is unique as a skeletal muscle that rhythmically contracts as a result of intrinsically generated action potentials [14] and displays structural characteristics similar to cardiac heart [10, 11]. Importantly, the avian lymph heart must be fully functional during *in ovo* development since its failure leads to oedema and embryonic death [10]. This is in sharp contrast to other skeletal muscles that remain relatively immature until after hatching. To our knowledge, no other studies have investigated how lymph heart becomes fully functional at early stages of the organism’s development.

Studies investigating electrical activity regulating the contraction of lymph hearts have mainly been performed in frog where the phenomenon was first documented by Bruck and Umrath [15], and later by Del Castillo and Sanchez [14]. Here, extracellular recordings via two platinum electrodes revealed that the lymph heart contraction was associated with bursts of electrical activity comprising 8–10 variable amplitude ‘*action currents*’ (local field potentials) [14]. Furthermore, intracellular recordings from lymph heart muscle cells revealed summating depolarisation followed by a later, sustained hyperpolarisation [14]. In addition, Del Castillo and Sanchez [14] and Day et al. [16]investigated pharmacological modulation of amphibian lymph heart electrophysiological properties to show that recorded activity was particularly sensitive to cholinergic modulators whilst subsequent studies revealed the presence of nicotinic acetylcholine receptors (nAChRs) in amphibian lymph heart [10, 17]. Thus, whilst the bioelectrical basis of the amphibian lymph heart contraction is well known and can be modulated via nAChRs in the same manner as skeletal muscle [10, 17], the underlying basis for avian lymph heart rhythmicity in birds remains unknown.

Here, we provide molecular evidence that avian lymph heart develops through an accelerated process in which events of skeletal precursor expansion, commitment and functionality occur concurrently together with the formation of the endothelial compartment. We postulate that hastened development is required so that the lymph heart acquires full functionality in a short period of time. Furthermore, investigation of electrical activity of lymph heart revealed that its excitability arises from an interplay of cholinergic receptors, voltage-dependent Ca^2+^ channels (L -type), intracellular Ca^2+^ and HCN channels. Although dynamics underlying rhythmicity may vary between avian lymph heart and cardiac pacemaker cells, these results show that similar molecular mechanisms mediate rhythmic contractile activity.

## Materials and Methods

### Whole-mount *in situ* hybridisation

Fertilised white eggs were obtained from Henry Stewart (UK) and incubated at 39°C and 80% humidity. Embryos at embryonic day (E) 6 (HH30), 8 (HH34), 10 (HH36) and 12 (HH38) were decapitated and cut transversally below the thorax. The caudal part of the embryo was skinned and fixed in 4% PFA for 12 hours or more. Dehydration in methanol (Fisher Scientific, Loughborough, UK) was then carried out and tissue stored at -20°C overnight. *In situ* hybridisation was performed as described previously [18] with the following digoxigenin-labelled probes: *MyoD* (1.5kb), *Myogenin* (1.2kb), *Pax-7* (582bp), *Cadherins* (*M* (*muscle*) (1.08kb), *N* (*neural*) (1.28kb), *R* (*retinal*) (1.6kb) and *T (Heart)* (931bp), *En-1*, *Prox-1*, L-type Ca^2+^ channels (*Cav 1*.*1;* 468bp), *HCN1* and *HCN4* channels. Following whole mount *in situ* hybridisation, embryos were fixed and photographed using a Nikon Coolpix digital camera and image processing was performed with Adobe Photoshop CS3 to adjust for brightness, contrast and background.

### Microelectrode arrays (MEA)

For MEA recordings, chick embryo pelvis was skinned at E10 (HH36), halved and one half of the pelvis used to ensure consistent contact of the intact lymph heart with the array of 60 electrodes. The pelvis was maintained in Kreb’s solution (containing 124mM NaCl, 3mM KCl, 1.25mM KH_2_PO_4_, 36mM NaHCO_3_, 1mM MgSO_4_, 10mM d-glucose and 2mM CaCl_2_)_._ Prior to recording, MEAs were cleaned with 5% (^w^/_v_) Terg-A-Zyme (Cole-Palmer, London, UK), and finally distilled water before air drying. Tissues were adhered to MEAs using a harp with lymph heart facing the bottom of the array. We used Nikon TS-51 microscope (Nikon, Japan) at magnification ×4 to ascertain the position of the lymph heart. Images of tissue and electrode positions were acquired using a Mikro-Okular camera (Meade Instruments Corp., CA, USA). Once attached, tissues were maintained at 25°C and were continually perfused with carboxygenated (95% O_2_/5% CO_2_) Kreb’s solution (∼2ml/min) and allowed to stabilize for at least 10 min prior to any recordings. Electrical activity across each tissue was monitored and recorded using MEAs (59 electrodes each 30μm diameter with 200μm spacing and 100μm recording radius. For stimulation, voltage pulses (STG2004, Multi-Channel Systems GmbH, Reutlingen, Germany) of 250 mV amplitude and 100 μs duration were applied with a 5s interval between pulses. Data acquisition was performed using MC Rack software (Multi Channel Systems GmbH, Reutlingen, Germany). Initially, tissue viability and contact with MEA electrodes was assessed by applying voltage pulses through MEA electrodes on the tissue. Once a reliable extracellular field potential had been obtained, 5 stimuli were applied and responses recorded for each electrode. Drugs were applied after recording reliable extracellular field potentials and were perfused for 20–30 minutes before re-applying the same stimulus-recording protocol. Signals were amplified (1200× gain), by a 120-channel dual headstage amplifier (MEA60 System, Multi-Channel Systems GmbH, Reutlingen, Germany) and simultaneously sampled at a minimum of 10 kHz per channel on all 60 channels. Once the recording was completed, the tissue still adhered onto MEA was kept at -20°C for 5 minutes and a final recording was made. Freezing kills the tissue and generates only a stimulus artefact in response to electrical stimulation, allowing offline subtraction of the stimulus artefact from each recording. Data analysis was performed using MC Rack (Multi Channel Systems GmbH, Reutlingen, Germany) and parameters such as maximum, minimum and peak-to-peak amplitude were extracted. The peak-to-peak amplitude of field potential was analysed for each electrode and data from individual electrodes were pooled to provide mean results for each lymph heart before artefact subtraction as described above. Drug effects were assessed by comparing peak-to-peak amplitude before and after drug application and results expressed as a normalised proportion of control values. Statistical significance was determined by non-parametric Mann–Whitney U-test in the case of all normalised data. In experiments where the effect of two drugs was tested, a non-parametric Friedman’s test with *post-hoc* Dunn’s test was used. In all cases p ≤ 0.05 was considered to be significant.

### Drugs

Nifedipine and tetrodotoxin (TTX) were obtained from Sigma–Aldrich (Poole, UK) and all other drugs were obtained from Tocris (Abingdon, UK). The concentrations of the drugs were as follows: atropine (5μM), carbachol (10μM), cyclopiazonic acid (CPA; 10μM), mibefradil dihydrochloride (10μM) nifedipine (20μM), TTX (5μM) and ZD7288 (4-**e**thylphenylamino-1, 2-dimethyl-6-m-ethylaminopyrimidinium chloride; 20μM). All drugs were dissolved in water except nifedipine (ethanol), CPA (DMSO) and TTX (citrate or acetate buffer at pH 4–5). Final bath concentration of vehicle did not exceed 0.01%.

### Optical recording of lymph heart beating

Chick embryo pelvis at E10 (HH36) were skinned and maintained in Tyrode’s solution (containing 145mM NaCl, 1mM Glucose, 5mM HEPES, 5.9mM KCl, 1.25mM CaCl_2_ and 1.2mM MgCl_2_.6H_2_0) on a heated stage at a temperature of 39°C or more to assess the beating of the lymph heart. The position of the lymph heart was ascertained by observation on a Nikon WD 70 microscope (Nikon, Japan) at magnification ×4 attached to Mikro-Okular camera (Meade Instruments Corp., CA, USA). Lymph heart contractions were recorded using ArcSoft WebCam Companion 2 (ArcSoft., CA, USA). Pharmacological investigations were not undertaken until a given lymph heart had exhibited robust beating for at least 5 minutes. 20μM of ZD7288 was then applied to determine the effect on lymph heart beating. The recording was then made continuously for 15–25 minutes until the lymph heart stopped beating due to the effect of drug administration. For quantification, video files generated were exported to Image J (NIH software) and beats were counted manually for the duration of the whole video. In addition, using Image J, a z-axis profile was plotted for lymph heart beating for each recording.

## Results

### Whole-mount *in situ* hybridisation results

**Fig 1:**
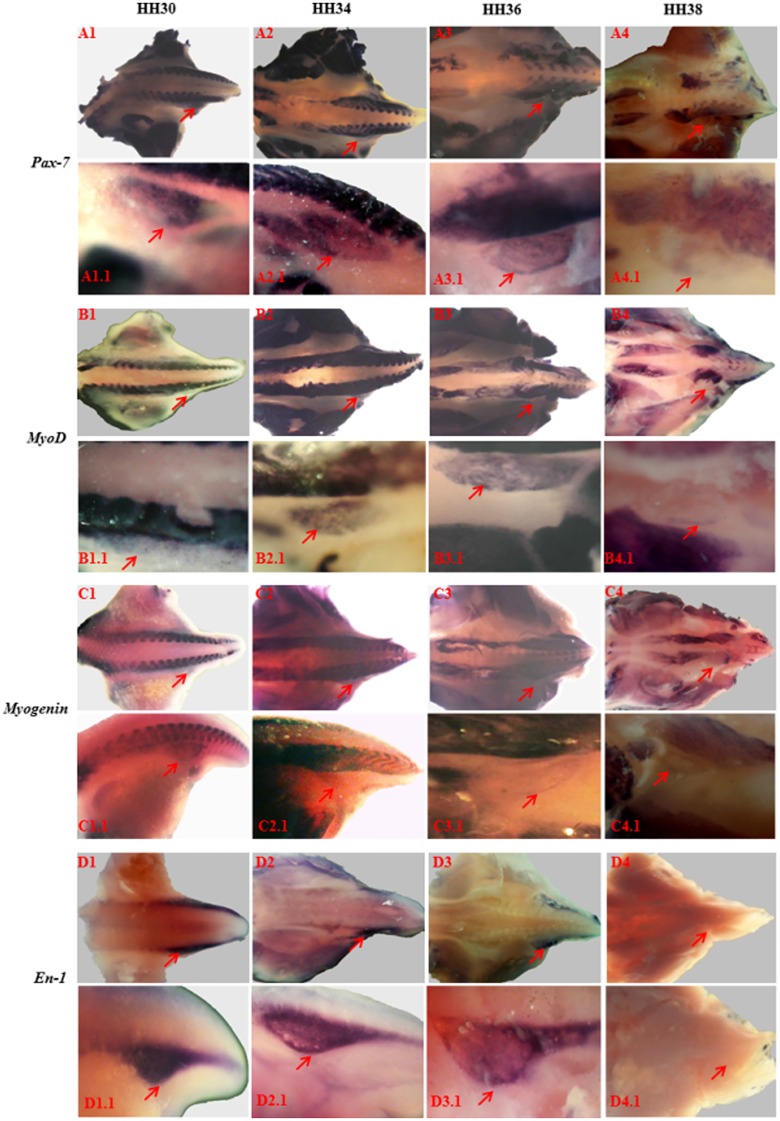
Expression of skeletal muscle markers during development of lymph heart. Whole mount in-situ hybridisation showing developmental expression of *Pax-7* (A1-A4), *MyoD* (B1-B4), *Myogenin* (C1-C4) and Engrailed-1 (*En-1*) (D1-D4) in the lymph heart as indicated by red arrow. The expression of *Pax-7*, *MyoD*, *Myogenin and En-1* was detected as early as HH30 and declined by HH38.

In order to understand the molecular mechanisms underpinning the development of the lymph heart, we first examined the temporal and spatial relationship between genes that control the early stages of muscle differentiation. During development of skeletal muscle, specifically limbs and trunk, myogenic precursors express members of the paired box family of transcription factors such as *Pax-3 and Pax-7* [19]. Commitment to myogenic lineage during development is signified by upregulation of *MyoD* and downregulation of *Pax*-*7*, and during terminal differentiation of myogenic precursors, *Myogenin*, is additionally expressed [20]. During lymph heart development, the expression of *Pax-7* was first detected at HH30 and, by HH38, its expression level was just detectable (Fig 1A1–1A4 and 1A1.1–1A4.1). It is noteworthy that during these stages, expression of *Pax-7* in axial and limb muscle was maintained at high levels (Fig 1A1–1A4). The expression of *MyoD* was detected at HH30 but**,** in contrast to *Pax-7*, its expression peaked at HH34 and, by HH38, had declined markedly (Fig 1B1–1B4 and 1B1.1–1B4.1). Surprisingly, the expression of *Myogenin* was detected as early as HH30 and was maintained until HH38 (Fig 1C1–1C4 and 1C1.1–4.1).

**Fig 2:**
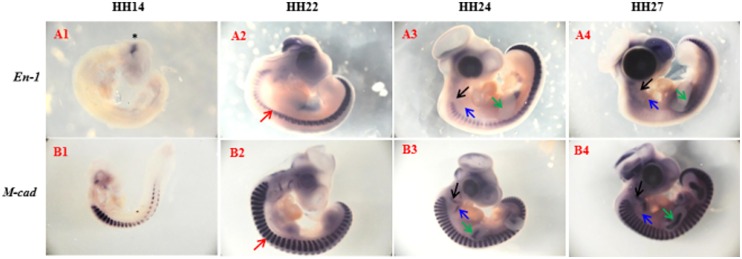
Expression of Engrailed-1 and M-cadherin during early avian development. Whole mount in-situ hybridisation showing developmental expression of *Engrailed-1 (En-1) (*A1-A4) and *M-cadherin (M-cad)* (B1-B4) at HH14, HH22, HH24 and HH27. At HH-14 *En-1* expression was only confined to the midbrain-hindbrain junction (indicated by asterisk (*) in A1). At HH22 (A2 and B2) and HH24 (A3 and B3), *En-1* expression is limited along the dorsal-ventral axis compared to M-cad (red arrow). Furthermore, *En-1* is not expressed in developing skeletal musculature of the head (black arrow), tongue muscle (blue arrow) and limb muscle (green arrow) (A3-A4). In contrast to *En-1*, *M-cad* is expressed in skeletal musculature of the head (black arrow), tongue muscle (blue arrow) and limb muscle (green arrow) (A4-B4).

Recent work has shown the development of amphibian lymph heart is dependent on the expression of *En-1* [11]. We therefore examined the expression of the avian homologue and found that, unlike *MyoD*, this gene was not expressed in developing limb or branchial arches (Fig 2A1–1A4). However, during development of lymph heart, *En-1* was highly expressed at HH30, HH34 and HH36 but, by HH38, its expression was not detectable (Fig 1D1–1D4 and 1D1.1–1D4.1). It is notable that expression of *En-1* had ceased in the epaxial region by HH30.

**Fig 3:**
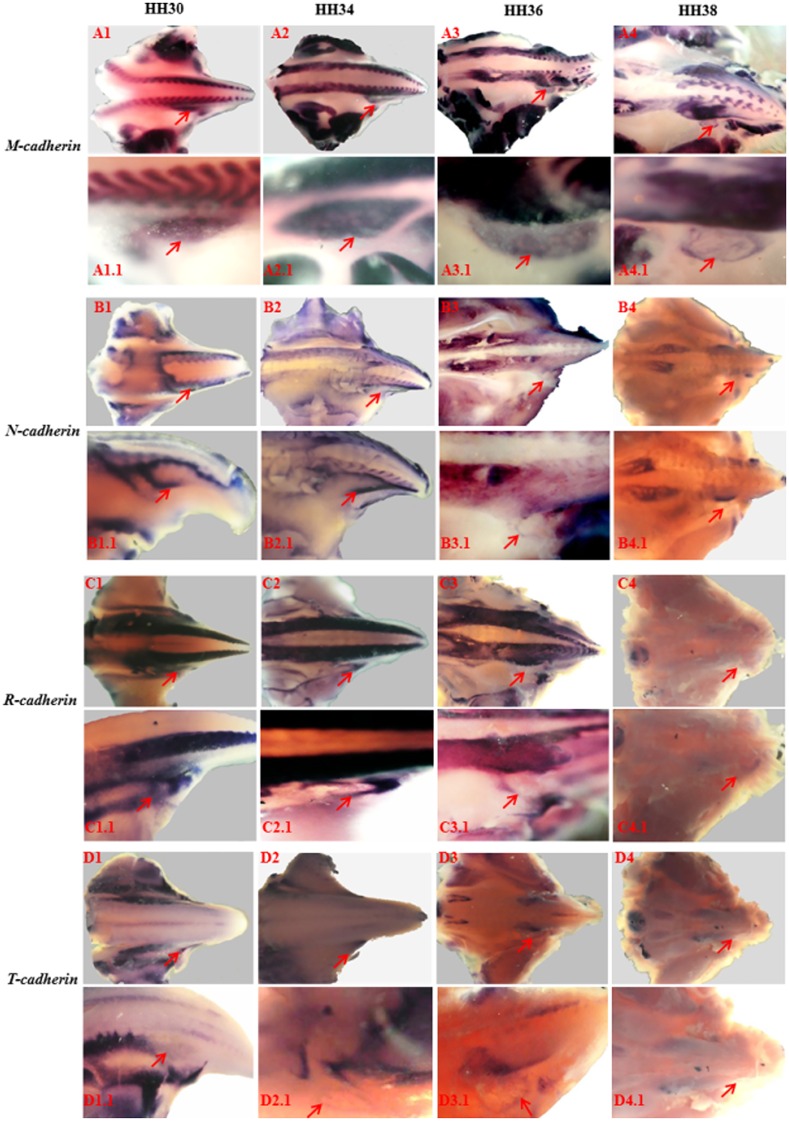
Expression of cadherins during lymph heart development. Whole mount in-situ hybridisation showing developmental expression of *M-cadherin* (A1-A4), *N-cadherin* (B1-B4), *R-cadherin* (C1-C4) and *T-cadherin* (D1-D4) in the lymph heart as indicated by red arrow. The expression of *M*, *N*, and *R*, and *T-cadherin* was detected as early as HH30 and only *M-cadherin* was expressed at HH38.

The expression of *cadherins* during lymph heart development was also assessed. *M-cadherin* is expressed in all muscle precursors (Fig 2B2–2B4). In the lymph heart, we found that *M-cadherin* was first detected at HH30 and by HH34 its expression was homogenous throughout the lymph heart. By HH38, its expression declined and was concentrated only at the border of the lymph heart (Fig 3A1–3A4 and 3A1.1–3A4.1). In most muscles the expression of other cadherins, especially *N****-*** and *R-Cadherin* are initiated after *M-cadherin*. Here, we found that the expression of *N****-*** and *R****-****cadherin* was confined to the outermost rim of the lymph heart with little or no expression in the centre of the lymph heart. The expression of *N****-*** and *R****-****cadherin* was detected as early as HH30, continued to be expressed until HH36 but, by HH38, expression of these *cadherins* was not detected (Fig 3B1–3B4 and 3C1–3C4). Conversely, *T-cadherin* was expressed throughout the lymph heart structure but, as with *N****-*** and *R****-****cadherin*, its expression was not detected at HH38 and its overall expression was markedly weaker than *M-cadherin* (Fig 3D1–3D4). Therefore, the cadherins also show a condensed programme of activation.

**Fig 4:**
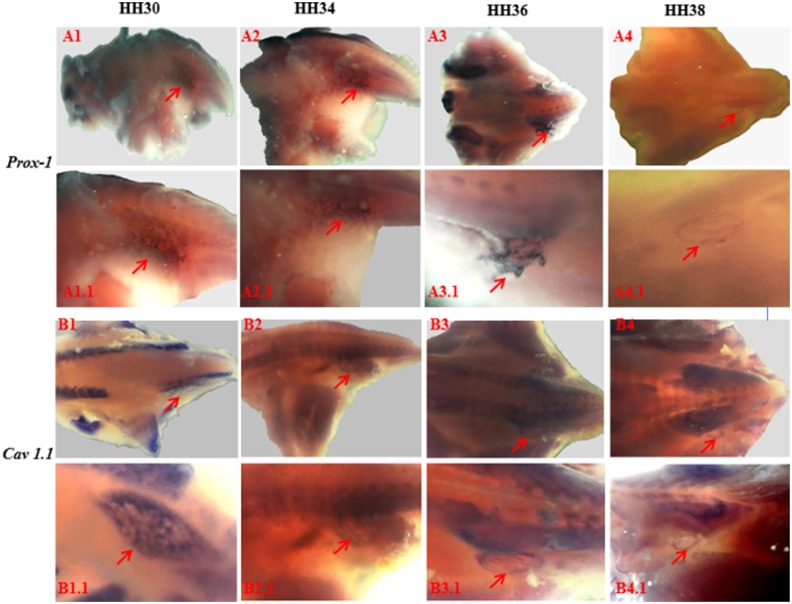
Expression of Prox-1, and Cav1.1 during lymph heart development. Whole mount in-situ hybridisation showing developmental expression of *Prox-1* (A1-A4) and *Cav1*.*1* (B1-B4) in the lymph heart as indicated by red arrow. The expression of *Prox-1* and *Cav1*.*1* was detected as early as HH30 and declined by HH38.

**Fig 5:**
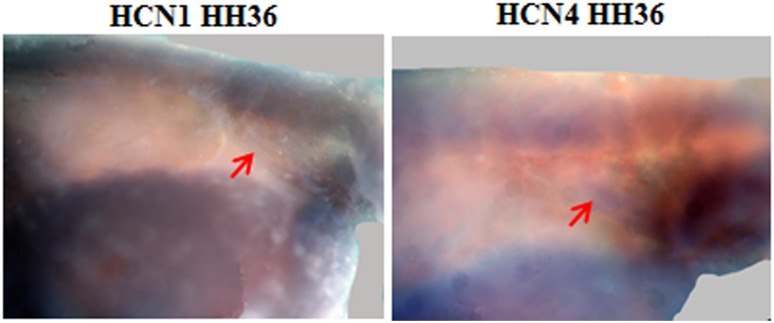
Expression of HCN1 and HCN4 at HH36. Whole mount in-situ hybridisation showing expression of *HCN1* and *HCN4* in avian lymph heart at HH36 as indicated by red arrow. The expression of these channels was not detected in other stages.

Furthermore, consistent with the lymphatic nature of the lymph heart, *Prox-1* was also expressed during development as reported previously **(**Fig 4A1–4A4) [11, 13, 21]. The expression of *Prox-1* was evident throughout the lymph heart and its expression was noticeable from HH30 and declined by HH38. We extended our study to investigate a marker for functional mediation of muscle activity by examining localisation of L-type voltage gated channels, in particular the *Cav1*.*1* subunit**,** in the lymph heart. Surprisingly, we found that *Cav1*.*1* was expressed at a very early stage of lymph heart development (HH30) and, by HH38, its expression was confined to the edge of the lymph heart (Fig 4B1–4B4 and 4B1.1–4B1.4). Moreover, we examined HCN channel expression and found that isoforms *HCN1* and *HCN4* were expressed only at HH36 and not in other stages (Fig 5). We also examined the expression of cardiac markers such as *GATA-4* (data not shown) and, consistent with results reported in amphibian lymph heart [11] did not detect any expression.

### Effect of ion channel blockers on extracellular field potential

**Fig 6:**
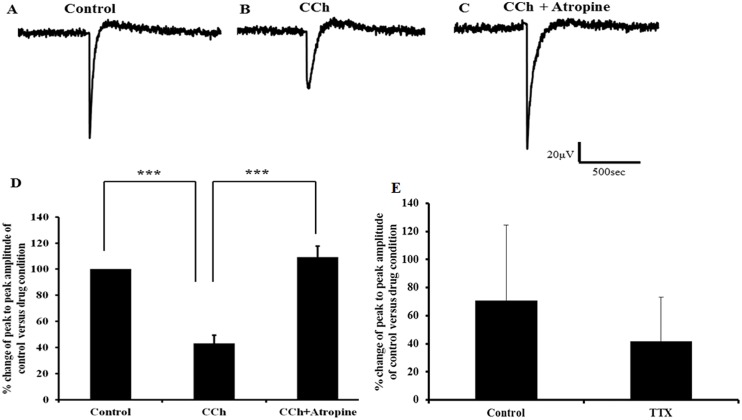
Evoked local field potential recorded from lymph heart before and after addition of carbachol followed by atropine in the presence of carbachol. A) Representative trace of evoked local field potential (LFP) obtained from control, in the presence of carbachol (10μM) B) and after adding atropine (5μM) in the presence of carbachol C). D) Bar charts show average peak to peak of evoked LFP amplitude calculated from control, in the presence of carbachol and after adding atropine in the presence of carbachol (n = 9). The data for evoked LFP in the presence of carbachol and the data for evoked LFP of atropine in the presence of carbachol was normalised to data obtained from control. After adding carbachol the average peak to peak of evoked **LFP** amplitude was significantly reduced (*** p<0.001) compared to the control. On addition of atropine in the presence of carbachol the effects were significantly reversed (*** p<0.001). Error bars represent mean ± SEM (n = 9). E) Bar chart shows average peak to peak of evoked LFP amplitude calculated from control, in the presence of TTX (n = 3). The data for evoked LFP in the presence of TTX was normalised to data obtained from control. After adding TTX the average peak to peak of evoked LFP was reduced compared to control however it was not statistically significant (p>0.05). Error bars represent mean ± SEM.

Our molecular profiling showed that the lymph heart predominantly expresses skeletal muscle markers and therefore we set out to investigate the contribution of ion channels responsible for excitation and contraction coupling in such tissue. Previous work has shown that the electrical activity of the lymph heart resembles skeletal muscle and the mechanical activity of the lymph heart is a result of the summation of bursts of acetylcholine-elicited postsynaptic potentials [16]. In the present study, consistent with the skeletal muscle nature of the lymph heart, the average peak to peak of the evoked local field potential (LFP) was significantly reduced to 43.1 ± 6.4% by the mixed mAChR and nAChR agonist, carbachol (10μM; p<0.001; Fig 6A–6D). Interestingly, subsequent addition of the mAChR antagonist, atropine (5μM) reversed the effects of carbachol, returning average peak-to-peak LFP amplitude to control levels (atropine: 109.3 ± 8.5%; p>0.05; Fig 6C–6D). This finding is consistent with results reported previously [16] which showed that the electrical activity of lymph heart is sensitive to cholinergic modulation. In addition, we also examined the effect of the sodium channel blocker, TTX where application appeared to reduce average peak-to-peak amplitude to 73.3 ± 31.0% of control (133 ± 118%) although this difference was not statistically significant (p>0.05), most likely due to the limited number of samples (n = 3; Fig 6E) and so necessitates future experiments to implicate any involvement of voltage-gated sodium channels.

**Fig 7:**
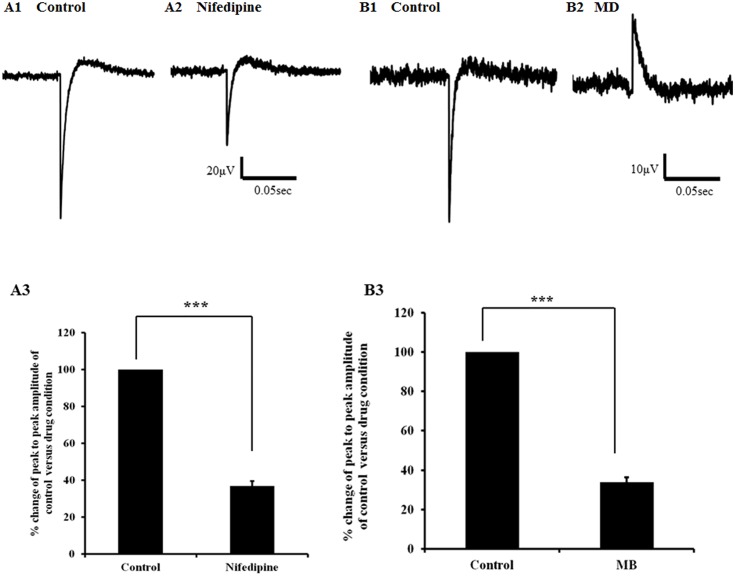
Evoked local field potential recorded from lymph heart before and after addition of nifedipine and mibefradil dihydrochloride. A1) Representative trace of evoked local field potential (LFP) obtained from control and in the presence of nifedipine (20μM) (A2). A3) Bar charts show average peak to peak of evoked LFP amplitude calculated from control and after adding nifedipine (n = 10). The data for evoked LFP in the presence of nifedipine was normalised to data obtained from control. After adding nifedipine the average peak to peak of evoked LFP amplitude was significantly reduced (*** p<0.001) compared to the control. Error bars represent mean ± SEM (n = 10). B1) Representative trace of evoked local field potential (LFP) obtained from control and after adding mibefradil dihydrochloride (MD) (10μM) (B2). B3) Bar charts show average peak to peak of evoked LFP amplitude calculated from control and after adding MD (n = 13). The data for the evoked LFP in the presence of MD was normalised to data obtained from control. After adding MD the average peak of evoked LFP amplitude was significantly reduced (*** p<0.001) compared to the control. Error bars represent mean ± SEM (n = 13).

As shown above, the L-type Ca^2+^ channel in particular *Cav 1*.*1* was expressed in the avian lymph heart. In order to determine the contribution made by L-type Ca^2+^ channels to lymph heart responsiveness, the effect of nifedipine (20μM), a selective blocker of voltage-gated L-type Ca^2+^ channel**s** [22], upon local field potentials evoked from lymph heart was investigated. Nifedipine significantly reduced the average peak-to-peak LFP amplitude to 36.9 ± 2.6% compared to control (p<0.001; Fig 7A1–7A3).

We next investigated ion channels that regulate rhythmicity such as T-type voltage-dependent Ca^2+^ channels and HCN channels. In order to determine the presence of T-type channels in avian lymph heart, mibefradil dihydrochloride (MD), a T–type voltage-operated Ca^2+^ channel antagonist, was used at a concentration of 10μM since higher concentrations can also inhibit L-type Ca^2+^ channels [23]. Although block of L type Ca^2+^ channels cannot be excluded, MD significantly reduced the average peak-to-peak evoked LFP amplitude to 33.9 ± 2.4% as compared to control (p<0.001; Fig 7B1–7B3). However, since the magnitude of the block observed was consistent with that seen following nifedipine treatment, these results show that voltage operated Ca^2+^ channels, most likely L-type, are important in generating contractile potentials in the avian lymph heart.

**Fig 8:**
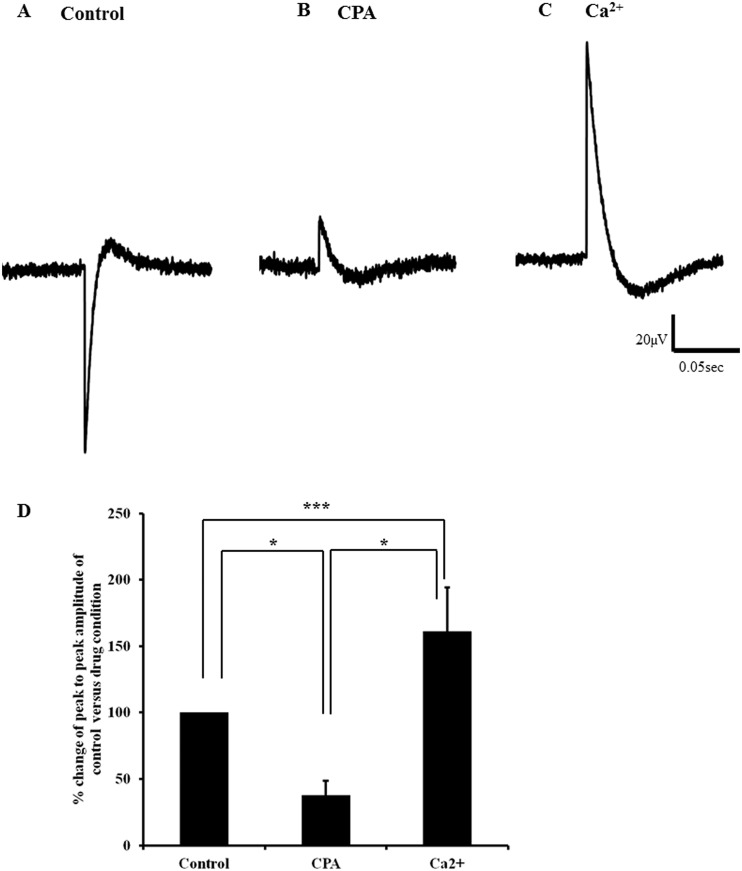
Evoked local field potential recorded from lymph heart before and after addition of cyclopiazonic acid followed by calcium. A) Representative trace of evoked local field potential (LFP) obtained from control, in the presence of CPA (10μM) B) and after adding Ca^2+^ (2mM) C). D) Bar charts show average peak to peak of evoked LFP amplitude calculated from control, in the presence of CPA and after adding Ca^2+^ (n = 13). The data for the evoked LFP in the presence of CPA and the data for the evoked LFP of Ca^2+^ was normalised to data obtained from control. After adding CPA the average peak to peak of evoked LFP amplitude was significantly reduced (* p<0.05) compared to control. On addition of Ca^2+^ the average peak to peak of evoked LFP amplitude significantly increased compared to CPA (*** p<0.001) and control (* p<0.05). Error bars represent mean ± SEM (n = 13).

Sarcoplasmic reticulum (SR) Ca^2+^ transport ATPase (SERCA) is the largest single contribution to Ca^2+^ removal in skeletal and cardiac muscle. To test the hypothesis that Ca^2+^ refilling is also crucial for the evoked potential seen in avian lymph heart, we recorded evoked LFPs first in Ca^2+^ free buffer and subsequently in the presence of the SERCA pump blocker, cyclopiazonic acid; (CPA; 10μM). As with nifedipine and MD, CPA significantly reduced the average peak-to-peak LFP amplitude to 37.8 ± 10.7% compared to control p<0.05) and addition of Ca^2+^ reversed the observed effect (161.4 ± 33.0%) where the average peak-to-peak LFP amplitude was not only significantly different from CPA (p<0.001) but also from control (p<0.05; Fig 8A–8D).

### Optical recordings of lymph heart beating

**Fig 9:**
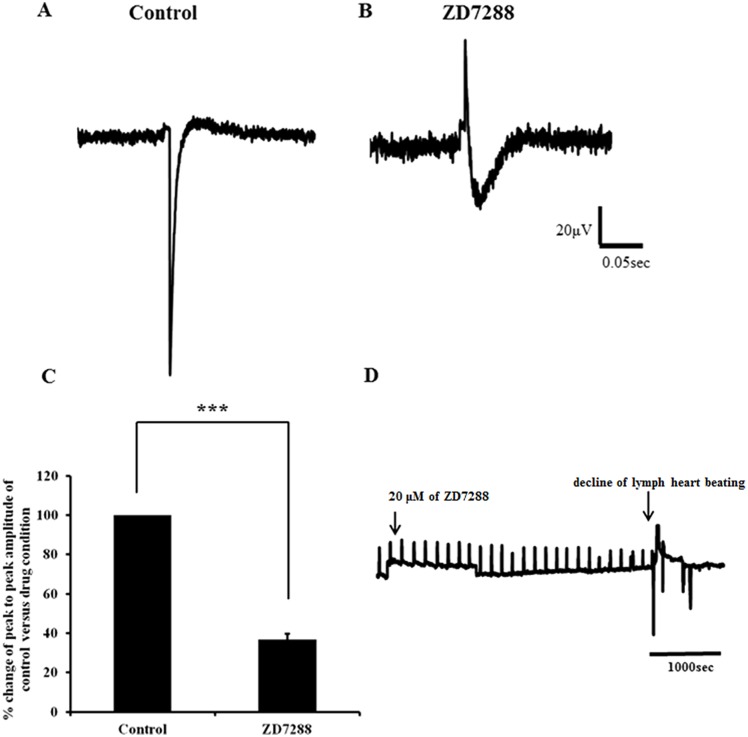
Evoked local potential recorded from lymph heart before and after addition of ZD7288 and effect of ZD7288 on lymph heart beating. A) Representative trace of evoked local field potential (LFP) obtained from control and in the presence of ZD7288 (20μM) (B). C) Bar charts show average peak to peak of evoked LFP amplitude calculated from control and after adding ZD7288 (n = 11). The data for the evoked LFP in the presence of ZD7288 was normalised to data obtained from control. After adding ZD7288 the average peak to peak of evoked LFP amplitude was significantly reduced (*** p<0.001) compared to the control. Error bars represent mean ± SEM (n = 11). D) Representative trace obtained by plotting z-axis profile of lymph heart beating. The arrow on the left indicates the time at which ZD7228 is added. The lymph heart beating declined with time and eventually stopped beating completely as indicated on the trace.

In order to complement the developmental expression profile presented here, we also examined the functional innervation of the lymph heart. Particularly, we looked at E10, the stage at which avian lymph heart exhibits spontaneous contraction [12]. In skinned pelvis (HH36), lymph hearts contract at a rate of 5 beats per minute on heated stage (39–40°C) as reported previously [10]. ZD7288 is widely considered a selective blocker of HCN currents [24]. After addition of ZD7288 (20μM), lymph heart continued to contract spontaneously at the same rate and after 10 min of drug administration the spontaneous contraction declined to a rate of 2 beats per minute in all samples examined (n = 6). After 15 minutes of drug administration, lymph hearts stopped contracting with no significant recovery observed after washing (Fig 9D). Furthermore, ZD7288 (20μM) significantly reduced the average peak-to-peak amplitude of evoked LFP to 38.6 ± 8.6% compared to control (p<0.001; Fig 9A–9C). These data show that the HCN channel is one of the candidates responsible for rhythmic contractions of the lymph heart.

Taken together, these results suggest that lymph heart expresses functional mAChRs and nAChRs in addition to a complement of ion channel**s** which have known involvement in excitation-contraction coupling and intrinsic rhythmicity.

## Discussion

### Musculature nature of lymph heart

Here, we examined the development of the avian lymph heart and electrophysiological properties using *in situ* hybridisation and MEA recordings respectively. Our molecular profiling showed that the avian lymph heart is similar to skeletal muscle but has a distinct expression profile of *Pax-7*, *MyoD* and *Myogenin*. We showed that *Pax-7* is expressed at the same time as *Myogenin* at HH30 and by HH38 there is a concomitant decline of *Pax-7* and *Myogenin*. During development of other skeletal muscles, the expression of precursor genes such as *Pax-7* is temporally and spatially separated from expression of markers of terminal skeletal muscle differentiation [25]. We also showed that *Pax-7* and *Myogenin* are co-expressed at early stage of HH30 in the lymph heart development. The co-expression, however, of *Pax-7* and *MyoD*, in the lymph heart is reminiscent of activated skeletal muscle cells [23]. From the expression profiles of *Pax-7*, *MyoD* and *Myogenin*, it appears that the process of myogenesis is not dynamic as is seen in skeletal muscle. Thus, in contrast to skeletal muscle, the processes of proliferation and differentiation do not appear to be mutually exclusive in lymph heart, suggesting that the development of the functional lymph heart may not necessarily be a subdivided process of proliferation and differentiation. We therefore suggest that the process of simultaneous proliferation and differentiation accelerates the development of the lymph heart, allowing it to become fully functional rapidly enough to successfully execute its essential role during *in ovo* life. The accelerated development of this skeletal muscle is also shown by the premature and overlapping expression of the cadherins and formation of the endothelial compartment (*Prox1*). We have previously shown that rapid transition from proliferation into differentiation is capable of accelerating development but has a major impact on muscle mass development by depleting the precursors required for sustained tissue growth [23] although we propose that this is not a risk factor in the avian lymph heart since it is only required for a short period of embryonic life.

Another important feature of avian lymph heart is the expression of *En-1*. Here we show that *En-1* is restricted to the epaxial region of dermomyotomes [26]. In zebra fish, *En-1* is expressed in the medial cells, known as adaxial cells, which subsequently migrate radially away from the notochord, becoming a superficial layer of muscle cells [25, 27]. Our *in-situ* hybridisation results showed that at HH30, *En-1* specifically marked lymph heart myoblasts and not any other type of myoblasts. We have shown previously that lymph heart develops from the hypaxial regions [10] and our results show that *En-1* is only expressed in the epaxial compartments during development suggesting that the expression of *En-1* represents a *de novo* specification event and not simply a trace from its somitic origin. Secondly, the expression of *En-1* is dependent on *Hedgehog* (*Hh*) signalling in *Xenopus* lymph heart and in the absence of Hh signalling and upon *En-1* knockdown, lymph heart muscle fails to develop and embryos consequently develop oedema [11]. Our results however, failed to detect expression of *Sonic Hedgehog* (*Shh;* data not shown) in avian lymph heart suggesting that the mechanisms that regulate *En-1* in avian lymph heart are different from the ones reported in *Xenopus* [11].

Muscle contraction is regulated by elevation of the intracellular Ca^2+^ concentration mediated by the interplay between two membrane proteins, the L-type voltage-gated calcium channels, VGCCs, and ryanodine receptors, RyRs. Here, we examined the expression of *Cav1*.*1* during development of the lymph heart and our results showed that the expression profile of *Cav1*.*1* is similar to skeletal muscle markers (*Pax-7* and *MyoD*). However, it was surprising that the expression of *Cav1*.*1* was evident in lymph heart at HH30. These results show that development of the lymph heart does not show the stepwise changes from precursor, to committed, to functionally active states which is seen during axial and limb development. Instead there is an accelerated process of development in which these events occur concurrently. We suggest that this hastened development is required in order for the lymph heart to become rapidly functional in a short space of time from its induction to the point at which it becomes redundant.

Interestingly, we also revealed expression of isoforms of HCN channels, namely HCN1 and HCN4. The expression of these channels coincides with the lymph heart exhibiting spontaneous contractions [12]. These two isoforms are also prominently expressed in the sino-atrial node suggesting that the mechanism underlying spontaneity of contraction in the lymph heart is similar to the blood heart. However, future studies examining blockade of HCN channels directly with siRNA may shed further light in the role of HCN channels and spontaneous activity of the lymph heart.

### Electrical activity of lymph heart

In our pharmacological investigations, the lymph heart was driven electrically in order to reduce variability in responsiveness commonly observed when recording bioelectrical activity in spontaneously beating organs. We examined electrically evoked local field potentials as a reliable indicator of the spontaneous activity in the lymph heart [14–16] to test the hypothesis that specific receptor types and ion channels regulate excitability of lymph heart. Carbachol, a parasympathomimetic drug that acts as an agonist at both muscarinic (mAChR) and nicotinic (nAChR) acetylcholine receptors, significantly reduced peak-to-peak evoked LFP amplitude; an effect that could be fully reversed by the mAchR antagonist, atropine consistent with previous reports [16]. Previous studies have showed that neuromuscular junctions at chick lymph heart muscle fibres exhibit acetylcholinesterase activity [10, 11] which diminishes as afferent innervation to the lymph heart degrades during late development [10]. Here, the role of lymph heart nAChR appear analogous to skeletal muscle as their stimulation causes muscular contraction. On the other hand, the mAChR acts as inhibitory cholinergic nerves similar to the vagal endings in the blood heart [28].

Pharmacological block of L-type Ca^2+^ channels significantly reduced peak-to-peak LFP amplitude and this channel is most likely responsible for excitation-contraction coupling in the lymph heart as has been reported for skeletal, smooth and cardiac muscle. Transmission electron microscopy has shown that, at E10, the muscular layer of lymph heart consisted of myoblasts containing largely immature contractile myofilaments with few Z disc striations [10] making it likely that, as with skeletal muscle, L-type Ca^2+^ channels in the avian lymph heart are also located in the T-tubules. In addition, in skeletal muscle, most Ca^2+^ that activates contraction originates from the sarcoplasmic reticulum and SERCA is considered to play a major role in Ca^2+^ removal during relaxation [29] 30. Our results showed that blocking the SERCA pump in the avian lymph heart with CPA significantly reduced peak-to-peak LFP amplitude. We propose that here, as with skeletal muscle and during excitation-contraction coupling of the lymph heart, L-type Ca^2+^ channels act as a voltage sensors with conformational changes leading to a physical interaction with ryanodine receptors (RyRs), promoting its opening, and triggering the release of Ca^2+^ from internal stores to cause a global Ca^2+^ transient. This leads to activation of SERCA which pumps Ca^2+^ back into the sarcoplasmic reticulum and inactivates RyRs. Future experiments to examine the expression of RyR1 and RyR2 are warranted and would provide greater insight on the role of ryanodine receptors during dynamic Ca^2+^ handling events in development of lymph heart. It should however be noted that the mechanism governing excitation-contraction coupling we describe here is the most simplistic and could be further modulated by the involvement of proteins such as calmodulin [30] and triadin [31], the potential role of which remain to be examined.

The mechanism described above for excitation-contraction coupling is insufficient to explain the rhythmic contractions seen in the lymph heart. Therefore, we also explored the potential contribution of other ion channels such as T-type Ca^2+^ channels and HCN channels. Since we cannot exclude the possibility that MD may also cause a block of L-type channels at the concentration used, we cannot conclude the exclusive involvement of T-type channels in the regulation of avian lymph heart rhythmicity although our results do implicate such involvement. Confirmation of our findings via investigation of the expression profile of T-type Ca^2+^ channels during development of lymph heart is warranted. Another candidate to explain the rhythmic contractions of the avian lymph heart is the HCN channel. The hyperpolarisation-activated current (I_h_) is a mixed cationic current carried by Na^+^ and K^+^ and recent evidence for small but significant Ca^2+^ permeability of these channels [32]. The role of HCN channels has been well documented in pace-making cells in the sinoatrial node and in the brain [33]. I_h_ is activated by hyperpolarisation to potentials negative to -50 to -60mV and inactivates upon depolarisation. Because membrane potential depolarises upon HCN activation, these channels can serve as pacemakers and modulate resting membrane potential and membrane properties of spontaneously active cells. Our results showed that HCN channels are responsible for the rhythmic contractions of the lymph heart as application of ZD7288, at 20μM to minimise off-target effects [34], irreversibly abolished lymph heart beating. Further support for the existence of HCN channels in modulating rhythmic contractions is provided by our MEA data where ZD7288 significantly reduced peak-to-peak LFP amplitude. While the precise localisation of these channels requires further investigation, it is known that among striated muscle fibres there are contracting-filament-fibres which resemble the bundle of His of blood heart in the lymph heart [33] making it likely that these channels are located in that area.

The presence of HCN channels in lymphatics has been reported previously, for instance, a study by McCloskey et al., [35] showed the presence of hyperpolarization-activated inward current similar to the one seen in sinoatrial node cells in the heart. Interestingly, this current was found only in 5% of the cells studied, suggesting the existence of specialized pacemaker cells in these vessels [35]. Further support for the existence of pacemaker cells in the lymphatics is provided by recent demonstration of a subpopulation of cells lying below the endothelium in mesenteric lymphatics. These cells show many of the immunohistochemical and ultrastructural features of the pacemaker cells [32].

## Conclusions

**Fig 10:**
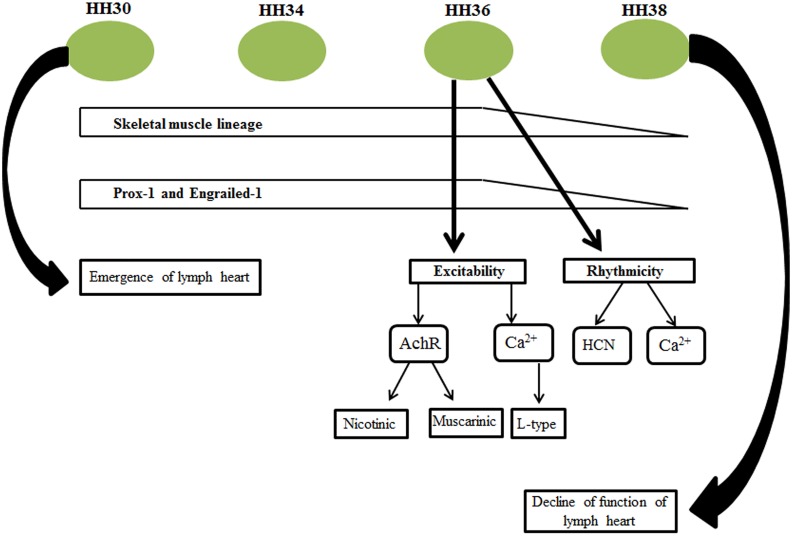
Summary of the genes regulated during lymph heart development and involvement of ion channels in excitability and rhythmicity of the lymph heart. The lymph heart predominantly expresses skeletal muscle markers and their expression is evident as early as HH30 and decline**s** by HH38. By HH36, the lymph heart contracts rhythmically and the basis for their underlying rhythmicity is most likely mediated by HCN channels while their excitation and contraction coupling is facilitated by cholinergic and L-type Ca^2+^ channels.

Our molecular data suggests that the developmental programme utilised by the avian lymph heart is suited for the rapid development of a functional organ at the expense of generating mass (Fig 10). Furthermore, we provide evidence based on *En-1* expression that it is homologous to the structure found in amphibians. Our microelectrode array results show that the avian lymph heart is sensitive to cholinergic and L-type Ca^2+^ channel blockers suggesting that cholinergic receptors and L-type Ca^2+^ channels are most likely important in excitation and contraction coupling. In addition, our results showed that HCN channels, contribute to maintenance of avian lymph heart rhythmicity. Therefore, the pathways implicated in regulating excitation and contraction coupling seem to operate independently of those regulating rhythmicity of the lymph heart. Taken together, these findings reveal unique insights into the developmental changes that give rise to lymph heart rhythmicity and so provide new avenues for experimental manipulation in cardiac bioengineering.
